# The proximate unit in Chinese handwritten character production

**DOI:** 10.3389/fpsyg.2013.00517

**Published:** 2013-08-09

**Authors:** Jenn-Yeu Chen, Rong-Ju Cherng

**Affiliations:** ^1^Department of Chinese as a Second Language, National Taiwan Normal UniversityTaipei, Taiwan; ^2^Department of Physical Therapy, National Cheng Kung UniversityTainan, Taiwan

**Keywords:** handwriting, Chinese, proximate unit, planning unit, word form encoding

## Abstract

In spoken word production, a proximate unit is the first phonological unit at the sublexical level that is selectable for production (O'Seaghdha et al., 2010). The present study investigated whether the proximate unit in Chinese handwritten character production is the stroke, the radical, or something in between. A written version of the form preparation task was adopted. Chinese participants learned sets of two-character words, later were cued with the first character of each word, and had to write down the second character (the target). Response times were measured from the onset of a cue character to the onset of a written response. In Experiment 1, the target characters within a block shared (homogeneous) or did not share (heterogeneous) the first stroke. In Experiment 2, the first two strokes were shared in the homogeneous blocks. Response times in the homogeneous blocks and in the heterogeneous blocks were comparable in both experiments (Experiment 1: 687 vs. 684 ms, Experiment 2: 717 vs. 716). In Experiment 3 and 4, the target characters within a block shared or did not share the first radical. Response times in the homogeneous blocks were significantly faster than those in the heterogeneous blocks (Experiment 3: 685 vs. 704, Experiment 4: 594 vs. 650). In Experiment 5 and 6, the shared component was a Gestalt-like form that is more than a stroke, constitutes a portion of the target character, can be a stand-alone character itself, can be a radical of another character but is not a radical of the target character (e.g., ± in 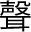, 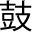, 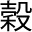, 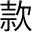; called a logographeme). Response times in the homogeneous blocks were significantly faster than those in the heterogeneous blocks (Experiment 5: 576 vs. 625, Experiment 6: 586 vs. 620). These results suggest a model of Chinese handwritten character production in which the stroke is not a functional unit, the radical plays the role of a morpheme, and the logographeme is the proximate unit.

In an alphabetic language such as English, to be able to write a word requires that language users know the alphabet (typically a small number of letters) and how to spell the word. The letters or graphemes serve as functional units in the orthography of a word (Van Galen, 1991; Kandel et al., 2011; Bonin et al., 2012). In a logographic language such as Chinese, to be able to write a word requires a different kind of knowledge. First of all, there is a need to distinguish a word from a character. A character can be a word, but a word usually consists of more than one character. Accordingly, it is the character that serves as the conscious target in the act of (hand) writing. Secondly, there are no letters and, accordingly, there is no equivalent of spelling in Chinese. In fact, it is not clear what the functional unit (analogous to a letter) is in the orthography of a character. This is why spelling does not readily apply in Chinese. Chinese users describe how to write, not spell, a character, and they do it in a combination of different ways. When a character is composed of components (radicals or simple characters) that bear names, Chinese users describe the character by naming each component and its position in the character. When there are nameless components in a character, Chinese users resort to strokes by attempting to name the strokes accompanied by laboriously writing the strokes in the air. These characteristics of Chinese characters as well as Chinese users' experience of describing how to write them point to the complexity of the issue about functional units in Chinese handwritten word production.

Traditionally, strokes are taken to be the functional units in the orthography of a Chinese character, and this makes some intuitive sense (Law et al., 1998). For example, in Chinese calligraphy, the eight strokes in the character 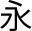 are considered the basic strokes that are representative of strokes in all characters such that mastery of these strokes guarantees success in writing all characters properly. Although calligraphy should not be equated with handwriting, this traditional wisdom serves to highlight the important role of strokes in the orthography of a Chinese character. Indeed, first-year writing lessons in Chinese often focus on strokes and their order in a character (Law et al., 1998). The Instructor's Manual of Standard Chinese Characters published by the Ministry of Education, Taiwan, lists 27–31 strokes, which can be characterized by 13 simpler stroke elements (http://www.edu.tw/files/site_content/M0001/std/fu.htm and http://dict.variants.moe.edu.tw/fulu/fu13/fubiau/bihua.htm). Strokes also feature prominently in psycholinguistic studies of Chinese reading (e.g., Yu and Cao, 1992; Peng and Wang, 1997; Taft and Zhu, 1997; Ding et al., 2004; Guo et al., 2005; Taft, 2006) as well as in studies of online recognition of Chinese characters (see Liu et al., 2006, for a review) as the number of strokes reflects the complexity of a character. Changizi and Shimojo (2005), considering strokes as the basic building blocks of characters, discovered some fundamental commonalities across all writing systems about how strokes combine to make characters and relate these to the constraints of the human visuo-motor systems. However, several studies have found that strokes do not seem to be the functional units in character recognition. For example, Chen and Yung (1989) observed no effect of stroke number on lexical decision. Similarly, Chen et al. (1996) observed no effect of stroke number on the simultaneous same-different comparisons of Chinese characters.

Many studies stressed the importance of radicals in the recognition of Chinese characters. Traditionally, radicals are components of characters that provide clues to the meaning of the characters. They are used for grouping and organizing characters in a dictionary. Contemporary psycholinguistic studies sometimes treat phonetic components as radicals and use the terms “radical” and “component” interchangeably. Taft and Zhu (1997) found that simple radicals are independently activated in the process of character recognition and they are position sensitive. Yeh and Li (2004) observed repetition blindness for characters that shared a radical, suggesting that radicals are functional units in word recognition. Ho et al. (2003) argued that the radical is an important orthographic processing unit in reading development in Chinese. None of these studies, however, speak against the role of strokes when they recognize the role of radicals.

Larger than a stroke but sometimes overlapping with a radical also finds a different unit of writing that seems to be functional. Chen et al. (1996) referred to it as the “stroke pattern.” Stroke patterns are those that “consistently and independently recur as integral constituents in composite Chinese characters” (p. 1026). For example, in the character 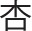 *xìng* “apricot,” 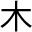 is a stroke pattern that also serves as a radical of the character, whereas, 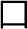 is just a stroke pattern. Both recur in other characters (e.g., 
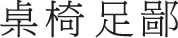
). Employing a speeded same-different comparison task involving Chinese characters, the authors found that response times were affected by the number of stroke patterns, but not by the number of strokes. They suggested that the integral stroke pattern is the functional orthographic unit in the recognition of Chinese characters, and that it is comparable to the letter in alphabetic word recognition.

Analyzing the writing errors of a dysgraphic patient, Law and Leung (2000) observed that many of these errors could not be categorized in terms of radicals. Rather, a unit larger than a stroke but smaller than a radical seemed to be involved. The authors referred to the unit as the logographeme. The logographemes are the smallest units in a character that are spatially separated and they appear in many characters, similar to the integral stroke patterns identified by Chen et al. (1996). Han et al. (2007) confirmed the psychological validity of the logographeme when they observed effects of frequency and number of logographemes in the writing performance of a dysgraphic patient. Based on normal and dysgraphic writing errors, Lui et al. (2010) proposed that the logographeme is the basic unit of Chinese writing and went on to establish a database of 249 logographemes based on the textbooks used in grade schools. Adopting a similar concept when analyzing Chinese characters, information technologists also established standards of Chinese character components for the purpose of digital processing by computers. For example, 560 logographemes are listed in the UCS Chinese Character Database (State Technology Supervision Bureau, 1994), 644 in the Hong Kong database of Basic Components of Kai-Font Characters (http://www.ogcio.gov.hk/tc/business/tech_promotion/ccli/cliac/glyphs_guidelines.htm).

Empirical support for the logographeme as the basic functional unit of Chinese handwriting has come primarily from error data. In the present study, we sought additional evidence from reaction time data in an online handwritten character production task. We examined and contrasted the roles of the stroke and the logographeme as the functional units in handwritten character production. The radical was also included in the study since it is by definition a logographeme, although it is not the smallest and it usually carries some semantic and phonological functions.

## The form preparation task and the experimental hypotheses

The handwritten character production task was adapted from the form preparation task that has been used extensively in spoken production research (Meyer, 1990, 1991). The original task involves having participants learn a set of prompt-target word pairs first, before cueing them to speak each target word upon seeing the associated prompt word. The to-be-spoken target words are arranged in a homogeneous block such that they share the initial phonological form (e.g., onset consonant). Or, they are arranged in a heterogeneous block such that the phonological forms are different across the target words. Reaction times for speaking the target words are typically faster when they appear in the homogeneous context than in the heterogeneous context, an effect commonly referred to as the form preparation effect and used to validate a functional unit. Afonso and Alvarez (2011) have adapted the task for handwriting, but their study concerned the issue of phonological activation in handwriting production and the language studied was Spanish. We adapted the task for handwriting in Chinese and investigated the issue of functional units in character production. The task asked participants to write the target characters upon seeing the prompt characters. Homogeneity was rendered in terms of shared orthographic form (e.g., initial stroke, initial logographeme, or initial radical). Based on previous research, we hypothesized that the radical must be a functional unit, although not the basic one, of Chinese character writing; therefore, we expected to observe a radical preparation effect. We hypothesized that the logographeme is the basic functional unit, and predicted a logographeme preparation effect. In contrast, we hypothesized that the stroke is not a functional unit, and predicted no stroke preparation effect. A total of six experiments were conducted. We report them together since they share the same method.

## Methods

### Participants

There were 12, 12, 24, 24, 20, and 18 participants in Experiments 1–6, respectively. The sample sizes varied due to the varying availability of participants at the time of the experiments. None of the participants participated in more than one experiment. They were all undergraduate or graduate students from National Cheng Kung University, Taiwan, volunteering for participation. They had normal or correct-to-normal vision with no known handwriting difficulties. Each was compensated 200 Taiwan dollars for their participation.

### Apparatus

The experiments were run on an IBM ThinkPad X60 laptop computer with a 12.1-inch color monitor or on an ASUS laptop computer with a 15-inch color monitor. A digital writing board with a pen (WACOM INTUOS 4 pen tablet) was connected to the computers to record participants' handwriting responses. The experiments were programmed in Visual Basic 2008.

### Materials and design

In each experiment, the materials consisted of 16 disyllabic words. The first character of each word served as the prompt while the second as the target. The words were arranged into four homogeneous sets of equal sizes such that the target characters within a set shared the first stroke (Experiment 1), the first two strokes that do not constitute a logographeme or a radical (Experiment 2), the first radical (Experiments 3 and 4), or the first logographeme (Experiments 5 and 6). The same words were also arranged into four heterogeneous sets of equal sizes such that the target characters no longer shared any initial orthographic forms. In both homogeneous and heterogeneous arrangements, the pronunciations of the target characters (syllables as well as tones) were all different. All the materials are listed in Table 1.

**Table 1:**
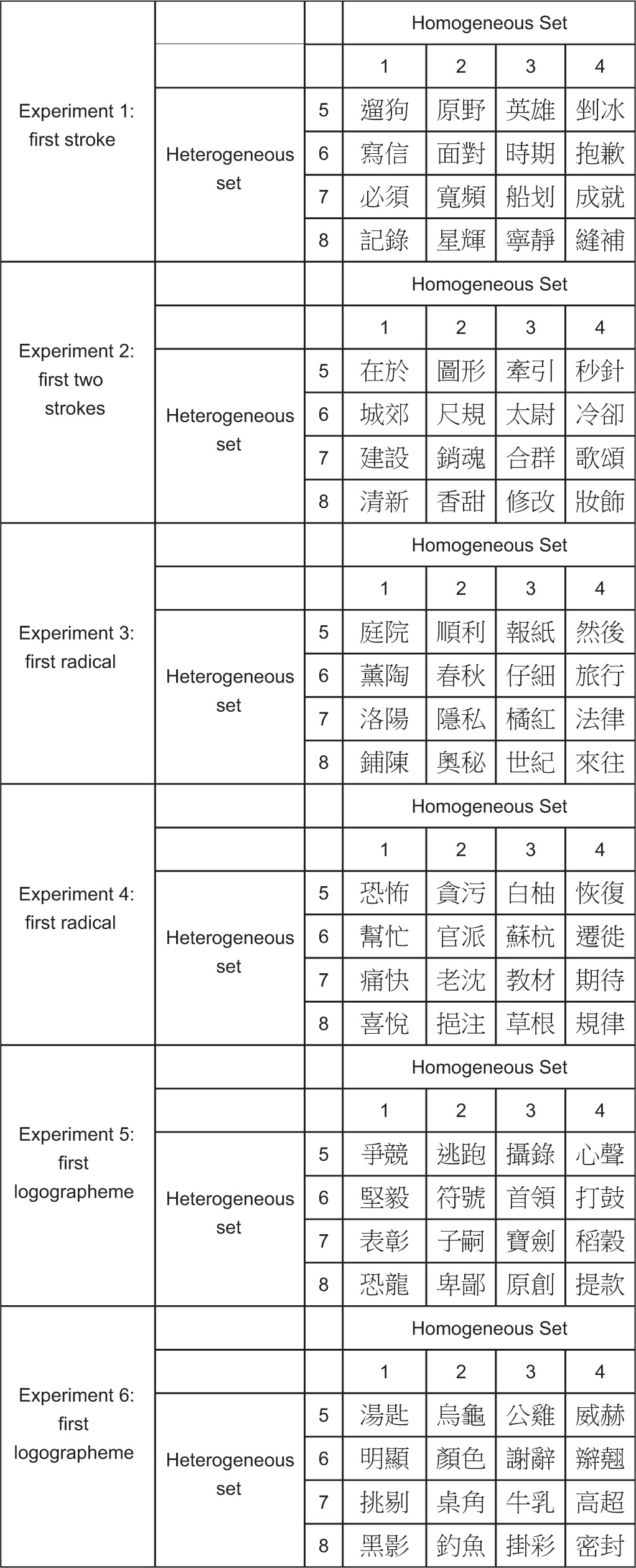
**Experimental materials used in each experiment**.

*The first character of each word served as the prompt while the second character as the target. Homogeneity is defined among the target characters*.

### Design and procedure

Each set of materials was delivered in a block. Before the experiment began, a practice set of four words (not overlapped with the experimental sets) were shown to the participants, who were told that they would be seeing the first character of each word one at a time and they were to write immediately, on the digital writing board, the second character of the word upon seeing the first. They were instructed to respond as quickly and accurately as possible and to write in the way they would usually write a word. The participants went through the practice set once in the same way they subsequently would the experimental sets. Before the delivery of each experimental set, the participants were always familiarized with the words in that set and reminded of what they would see and should respond.

The participants always completed the homogeneous or the heterogeneous sets before receiving the other sets. The order of the two kinds of sets was counterbalanced across the participants. The four homogeneous sets were randomized and administered twice; so were the four heterogeneous sets. Within a set, each word was repeated four times so that there were 16 trials, the order of which was random.

On each trial, two dashes appeared on each side of a blank window at the center of the screen and lasted for 200 ms. The dashes were replaced by the prompt character for 600 ms. As soon as the prompt character appeared, the participants wrote the target character the way they were instructed. Their writing appeared simultaneously on the screen below the prompt character. Response times were measured in milliseconds from the appearance of the prompt to the time when the digital pen touched the writing board. The participants had to initiate a response within 1500 ms from the onset of the prompt or else the trial was terminated and recorded as “no response.” A 2500-ms response period was allotted for completing the writing. The written character was recorded and stored as an image file for subsequent checking of accuracy. The participants then pressed the spacebar to proceed to the next trial. Each experiment took about 1 h to complete.

Errors were coded to include no responses, false starts (beginning with an incorrect stroke and correcting it before completing that stroke), immature starts (pen touching the board before actual writing), and wrong responses. They ranged from 1 to 3% across experiments (see Table 2). Outliers, defined as the data points falling outside the range of mean plus and minus 3 standard deviations, were first removed from the response times of correct trials before statistical analyses. The main factor in the experiments was type of stimulus sets (homogeneous or heterogeneous), which was a within-participants factor; so was repetition. Order of type of sets was a between-participants factor.

**Table 2:** **Means and standard errors (in parentheses) of the response times (in ms) for the homogeneous and the heterogeneous sets, as well as the preparation effects computed as the differences of the heterogeneous RTs minus the homogeneous RTs**.

	**Homogeneous**	**Heterogeneous**	**Effect**
Experiment 1: First stroke	687.0 (10.3) 1%	683.6 (7.9) 1%	−3.4
Experiment 2: First two strokes	716.6 (9.5) 2%	715.7 (11.3) 3%	−0.9
Experiment 3: First radical	685.2 (8.5) 2%	704.1 (7.6) 2%	18.9^*^
Experiment 4: First radical (replication)	593.7 (9.9) 1%	650.5 (6.1) 1%	56.8^*^
Experiment 5: First logographeme	575.9 (11.5) 2%	625.5 (9.5) 2%	49.6^*^
Experiment 6: First logographeme (replication)	585.9 (8.8) 3%	620.0 (6.7) 3%	34.1^*^

*The percentages are mean error rates*.

* *The effect is significant at *p* < 0.0001*.

## Results

Table 2 presents the mean response times of correct trials for different types of sets and for each experiment. Preparation effects were computed as the differences of heterogeneous RTs minus homogeneous RTs. They are presented in the last column of the table. As the table shows, there was no preparation effect when the target characters shared the first stroke (Experiment 1). This was the case even when two initial strokes were shared (Experiment 2). By contrast, there was a large preparation effect when the target characters shared the first radical (Experiments 3 and 4) or the first logographeme (Experiments 5 and 6).

The response times were analyzed with the linear mixed model using the Mixed Procedure of Statistical Analytic System (SAS), with both participants and items treated as random factors. For experiment 1, the critical factor, type of stimulus sets, was not significant: *F* = 0.47, *p* = 0.49, confirming the absence of a preparation effect. Repetition was significant: *F* = 402.36, *p* < 0.0001, but repetition X type of stimulus sets was not: *F* = 0.60, *p* = 0.44. The order of stimulus sets was not significant: *F* = 1.29, *p* = 0.25, although the interactions involving this factor were, *p*'s < 0.0001.

For Experiment 2, the critical factor, type of stimulus sets, was not significant: *F* = 0.03, *p* = 0.85, confirming the absence of a preparation effect. Repetition was significant: *F* = 264.26, *p* < 0.0001, but repetition X type of stimulus sets was not: *F* = 0.59, *p* = 0.44. The order of stimulus sets was significant: *F* = 4.45, *p* < 0.04; so was the interaction of order and type of stimulus sets: *F* = 229.52, *p* < 0.0001. Order of stimulus sets did not interact with repetition, *F* = 0.02, *p* = 0.89, although the three-way interaction involving type of stimulus sets, order of stimulus sets and repetition was significant, *F* = 50.01, *p* < 0.0001.

For Experiment 3, the critical factor, type of stimulus sets, was significant: *F* = 55.45, *p* < 0.0001, confirming the presence of a preparation effect. Repetition was significant: *F* = 551.78, *p* < 0.0001, and repetition X type of stimulus sets was too (at borderline): *F* = 3.71, *p* = 0.054. When the effect of type of stimulus sets was examined separately for each repetition, it was significant in both: *F*'s = 43.9and 17.3, *p*'s < 0.0001. The order of stimulus sets was not significant: *F* = 0.10, *p* = 0.75. Neither was the order of stimulus sets X repetition interaction: *F* = 1.92, *p* = 0.16. However, type of stimulus sets interacted with order of stimulus sets: *F* = 150.89, *p* < 0.0001. The three-way interaction was also significant: *F* = 88.63, *p* < 0.0001.

For Experiment 4, the critical factor, type of stimulus sets, was significant: *F* = 597.98, *p* < 0.0001, confirming the presence of a preparation effect. Repetition was significant: *F* = 312.62, *p* < 0.0001, and so was the repetition X type of stimulus sets interaction: *F* = 4.67, *p* < 0.01. When the effect of type of stimulus sets was examined separately for each repetition, it was significant in both: *F*'s = 91.3 and 114.1, *p*'s < 0.0001. The order of stimulus sets was not significant: *F* = 0.00, *p* = 0.99, although its interactions with other factors were: *p*'s < 0.0001.

For Experiment 5, the critical factor, type of stimulus sets, was significant: *F* = 197.91, *p* < 0.0001, confirming the presence of a preparation effect. Repetition was significant: *F* = 902.42, *p* < 0.0001, but the repetition X type of stimulus sets interaction was not: *F* = 0.56, *p* = 0.45. The order of stimulus sets was not significant: *F* = 0.00, *p* = 0.95, although its interactions with other factors were: *p*'s < 0.01.

For Experiment 6, the critical factor, type of stimulus sets, was significant: *F* = 139.36, *p* < 0.0001, confirming the presence of a preparation effect. Repetition was significant: *F* = 580.76, *p* < 0.0001, and so was the repetition X type of stimulus sets interaction: *F* = 4.53, *p* = 0.03. When the effect of type of stimulus sets was examined separately for each repetition, it was significant in both: *F*'s = 42.9 and 115.9, *p*'s < 0.0001. The order of stimulus sets was not significant: *F* = 1.58, *p* = 0.21, although its interactions with other factors were: *p*'s < 0.0007.

To summarize, the most important results of the statistical analyses across experiments are that the critical factor, type of stimulus sets, was significant when the target characters shared the first radical or the first logographeme, but not when they shared the first stroke or the first two strokes. When the effect of type of stimulus sets was non-significant, it did not vary across repetitions. When the effect of type of stimulus sets was significant, it tended to vary with repetitions, but only in the relative size of the effect; in both repetitions the effect was significant. Any interaction of type of stimulus set with order of stimulus set is expected but does not concern us. The latter was included in the design precisely for the purpose of counterbalancing a potential order effect.

## Discussion

Research on the functional unit of a character in Chinese handwritten production is scarce, and draws evidence primarily from patient data. The present study investigated the functional unit of a Chinese character when it is being written by normal adults. Three levels of unit were examined: stroke, logographeme, and radical. Six experiments employing the handwritten version of the form preparation task found significant preparation effects for the radical and the logographeme, but no preparation effect for the stroke. Sharing a single stroke did not produce a preparation effect; neither did sharing two strokes.

Testing for an effect of sharing two strokes (Experiment 2) is important because the effect for sharing one stroke may be too small to be detectable, and sharing two strokes might give the stroke an increased chance of demonstrating its functional role. The lack of a preparation effect for sharing two initial strokes in Experiment 2 serves to strengthen the evidence that the stroke is not a functional unit in Chinese handwritten character production.

Although the radical produced a preparation effect, it varied greatly in size between experiments. It is not clear what factors have brought about the variability. An examination of the radicals tested in the two experiments reveals that they tend to be more complex (in terms of number of strokes and perhaps type of strokes) in Experiment 3 than in Experiment 4. As radicals can vary greatly in complexity, future research needs to investigate how complexity affects the preparation of a radical in handwritten production.

The preparation effects for the logographeme also varied between experiments, but much less than the preparation effects for the radicals. This may be taken as evidence that the logographeme is more basic a functional unit than the radical in Chinese handwritten production.

The relationship between the logographeme and the radical may be understood by an analogy to the letters and morphemes in English. A logographeme is analogous to a letter or grapheme in English. It is the building block of a Chinese character. A radical, by carrying some semantic or phonological function, is analogous to a morpheme in English. However, it should be kept in mind that a radical as the morpheme of a character represents a different level of representation than a character as the morpheme of a multi-character word. The stroke in the Chinese orthography plays the same role and function as the stroke in the English orthography.

### A tentative model of chinese handwritten character production

In this section, we propose a tentative model of Chinese handwritten character production based on our findings as well as the assumptions described in the last paragraph. The model adopts the same architecture as that of the spoken production model postulated by Levelt et al. (1999). The model needs to specify explicitly the processing mechanisms as well as the processing units (i.e., functional units) of handwriting. Although a number of handwritten production models are available and meet our needs (e.g., Van Galen, 1991; Rapp and Caramazza, 1997; Kandel et al., 2011; Bonin et al., 2012), we chose to adapt the Levelt et al.'s spoken model because the existing models of handwritten word production bear overall resemblance to the model of spoken word production and because Levelt et al.'s model contains more processing details with respect to word form encoding and has been extensively tested across languages (including Chinese, cf. Chen et al., 2002; Chen and Chen, 2006, 2007, 2013) in recent years with the form preparation task which we used for our study.

The spoken production model postulates a stage of morphological encoding during which a morphologically complex word is first decomposed into its constituent morphemes together with the morphological frame of the word. The next stage, phonological encoding, takes each decomposed morpheme as input and retrieves its phonological contents (segments and metrical frame). This is followed by the incremental, left-to-right assignment of the segments to the corresponding slots in the frame according to the language-specific phonotactic principles (a process called prosodification/syllabification), and results in a phonological word (in the form of linearized phonological syllables) for subsequent stages of processing.

**Figure 1:**
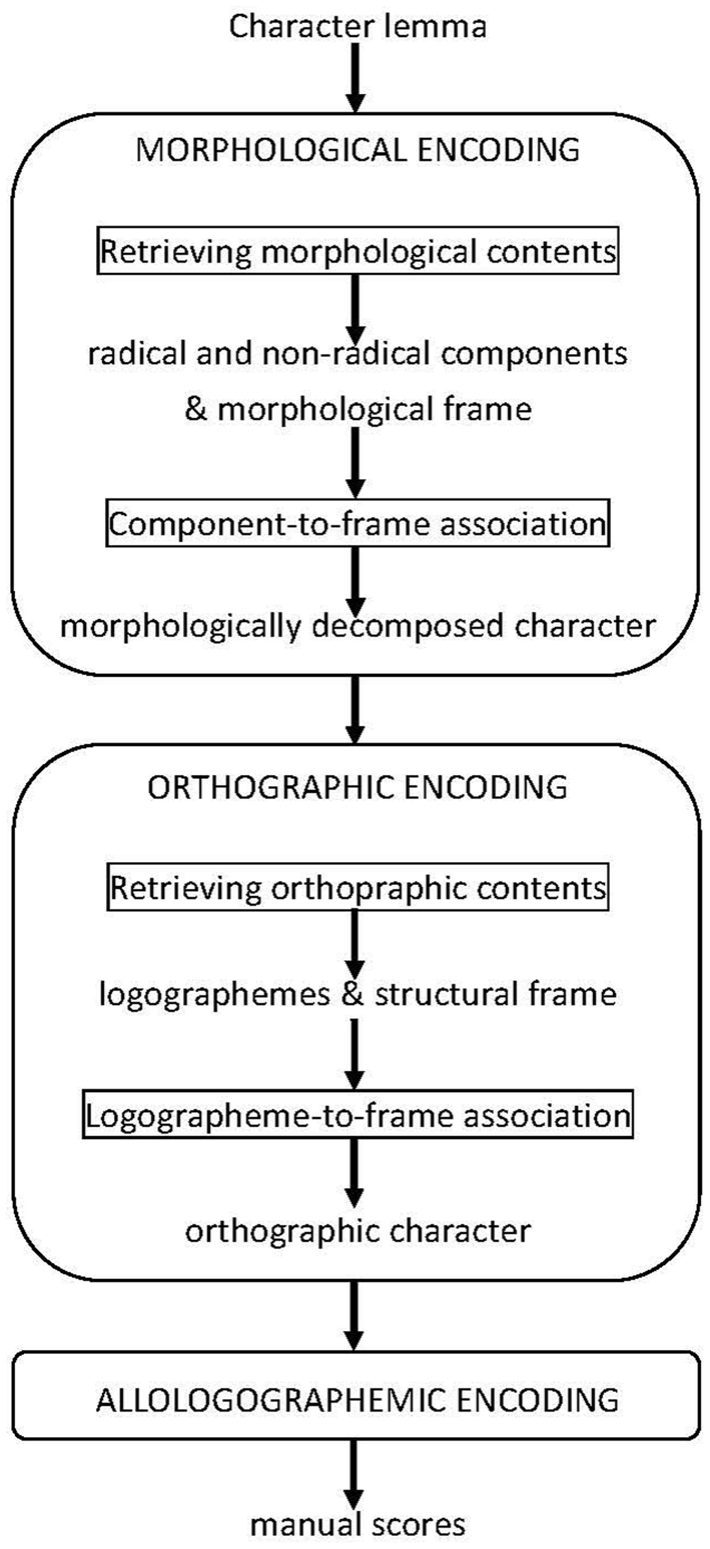
**A tentative model of Chinese handwritten character production**.

To adapt the spoken model for Chinese handwritten production (see Figure 1), we focus on the character rather than the word. As explained earlier, this is because the character is usually the conscious target of handwriting in Chinese. We focus further on morphologically complex characters rather than morphologically simple ones. Simple characters are single perceptual wholes (e.g., 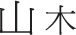), while complex characters are made up of separable perceptual wholes (e.g., 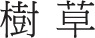). Complex characters fall into morphological families defined by radicals (e.g., 
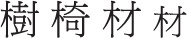
 etc., belong to the 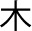 “wood” family). The handwritten character production model makes the parallel processing assumptions to the spoken model. A complex character is first decomposed into simple character forms that correspond to the radical and the non-radical components (or morphemes). A morphological frame is also spelled out along with the morphemes. The morphemes contain position and order codes which determine how they are assigned to the slots in the frame. During the (next) stage of orthographic encoding, the orthographic contents of each morpheme are retrieved which consist of the logographemes as well as the structural frame of the morpheme. These units are then inserted sequentially into the slots in the frame according to the orthotactic principles of Chinese. The result is a square-shaped orthographic form (called orthographic character) to be taken up by the next stages for allologographemic encoding and motoric processing. These last stages are included just to complete the processing and are not specified with any detail.

The Chinese handwritten production model also inherits the suspension-resumption mechanism from the original spoken model. The mechanism allows the word form encoder of the production system to proceed as far ahead as possible to prepare the beginning portion of a character if it is known. The encoder then suspends operation and resumes it only when the remaining portion of the character becomes known. Under this model, the portion of a character that can be prepared in advance is, by definition, a functional unit or made up of functional units. Stated in a different way, a functional unit is the part of a character that has a mental representation in a writer's orthographic lexicon, and is accessed and sequentially ordered when the writer prepares to write the character. In the model, both the radical and the logographeme are functional units, but they are different levels of representation and are processed at different stages. The stroke is not considered a functional unit, the way a function unit is conceived in this model. For spoken word production, O'Seaghdha et al. (2010) recently proposed the concept of proximate unit to capture the first phonological unit at the sublexical level that is selectable for production. Adopting the concept and applying it to handwritten production, we propose the logographeme is the proximate unit in Chinese handwritten character production.

Although the logographeme is considered the proximate unit in Chinese handwritten character production, the current definition of a logographeme is subject to operational ambiguity. Lui et al. (2010) offered a list of logographemes, but did not tell us how to decompose a character into its composing logographemes. The China National Commission for Language, and Script (1997) established a standard of character components for the GB13000.1 character set. But the criteria appear complex and opaque. Moreover, it was created for the purpose of facilitating information processing of Chinese characters by computers. Whether these components are also represented and processed in the human users remains unknown and requires extensive psycholinguistic research.

The tentative model proposed herein contains sufficient processing details and assumptions, which can lead to hypotheses for further investigations. For example, the postulation of a separate stage of morphological encoding needs to be scrutinized. Because a radical also fits the definition of a logographeme, perhaps it is represented and processed in the same way as non-radical logographemes and there is no need to postulate a separate stage of morphological encoding. Indeed, past research has analyzed Chinese characters into distinct structures, which are not particularly defined around the radicals (e.g., Yeh and Li, 2002, 2004). It is conceivable that these structures are structures for organizing the logographemes. On the other hand that a character consists of a morphological structure seems to be widely assumed in the recognition literature, and a radical does carry a semantic function that makes it morphological. The work for future research may be to determine if the radical plays a special role above and beyond that of the logographeme.

### Conflict of interest statement

The authors declare that the research was conducted in the absence of any commercial or financial relationships that could be construed as a potential conflict of interest.
